# Evaluation of Performance of Functionalized Amberlite XAD7 with Dibenzo-18-Crown Ether-6 for Palladium Recovery

**DOI:** 10.3390/ma14041003

**Published:** 2021-02-20

**Authors:** Oana Alexandra Grad, Mihaela Ciopec, Adina Negrea, Narcis Duteanu, Petru Negrea, Raluca Vodă

**Affiliations:** Faculty of Industrial Chemistry and Environmental Engineering, Politehnica University of Timişoara, 2 Piata Victoriei, RO 300006 Timisoara, Romania; oana.grad@upt.ro (O.A.G.); adina.negrea@upt.ro (A.N.); narcis.duteanu@upt.ro (N.D.); raluca.voda@upt.ro (R.V.)

**Keywords:** palladium, recovery, adsorption, crown ethers, Amberlite XAD7

## Abstract

Due to the increased demand for palladium, as well due to its reduced availability in nature, its recovery from diluted waste solutions becomes a necessity, and perhaps an emergency. As a result of economic and technological development, new materials with improved adsorbent properties that are more efficient for metallic ions’ recovery were synthesized and introduced to market. The goal of this study was to obtain a new adsorbent material by functionalizing through impregnation a commercial polymeric support that was both inexpensive and environmentally friendly (Amberlite XAD7) with crown ether (di-benzo-18-crown-6—DB18C6). Crown ethers are known for their ability to form complexes within metallic ions, by including them inside of the ring, regardless of its atomic size. Adsorbent material was prepared by impregnation using the solvent-impregnated resin method (SIR). To highlight the presence of crown ether on the resin surface, a new synthesized material was characterized by scanning electron microscopy (SEM), elemental analysis X-ray energy dispersive spectroscopy (EDX) and Fourier transform infrared spectroscopy (FT-IR). The specific surface of the adsorbent material was also determined by the Brunauer–Emmett–Teller (BET) method. Adsorbent performances of the prepared material were highlighted by kinetic, thermodynamic and equilibrium studies and a possible mechanism was also proposed. The influence of specific parameters for the adsorption process (contact time, temperature, Pd(II) initial concentration) on the maximum adsorption capacity was pursued.

## 1. Introduction

An important class of elements it is represented by platinum group metals (PGMs)—i.e., iridium, palladium, osmium, rhodium and ruthenium—due to their large range of applications in various industries [1]. Like all noble metals, palladium possesses attractive physical and chemical properties such as high melting point, good corrosion resistance and remarkable catalytic properties [2,3]. Considering all these properties, palladium has been a vital material in industries such as refining, petrochemical, medicine, jewelry and ornaments, electronic and electrical industries, integrated circuits, systems with good corrosion resistance, dental alloys and as a catalyst for different industrial processes [2].

Because of all the benefits of Pd usage, technological development has led to a significant growth in the demand for Pd, which almost inevitably led to an increase of Pd release into the environment. Such environmental release causes the contamination of food and waters worldwide [2,3], leading finally to bioaccumulation in different living organisms [3]. Experimental data proved that Pd has no biological role, being at the same time highly toxic and carcinogenic to humans, causing asthma, rhino-conjunctivitis, allergies, etc. [3,4]. In this context, due to the limited resources, it is very important to find a way to recover Pd from wastewaters produced by different industries which use PGMs extensively [2,4,5,6]. Pd recovery and recycling becomes feasible by developing new techniques for Pd effective pre-concentration and separation from such wastewaters [2].

Recently, adsorbent materials were developed by functionalization of halloysite nanotubes with amino groups, and further, such materials were used for Pb^2+^ ions’ removal [7]. Nanocomposite materials based on clay nanoparticles were developed and used for removal of inorganic and organic pollutants [8].

The main technologies used for PMG recovery during recent decades have been represented by solvent extraction, precipitation, membrane separation [1] and ion exchange technologies [6]. In case of conventional recovery techniques, extraction and precipitation from different waste liquids involve usage of different synthetic reagents with different adsorption capacities/selectivity. In case of wastewaters containing low concentration of Pd(II) ions, such treatment technologies present a large disadvantage: low efficiency, incomplete metal recovery, high capital cost and high complexity—involving large-scale equipment and large amounts of effluents and chemicals. These technologies are responsible for generating large amounts of secondary wastes [1]. From all recovery methods used today, adsorption attracts great interest due to its high selectivity for different metallic ions and because it also depends on the nature of the used adsorbent material.

Therefore, interest in obtaining new materials with good adsorbent properties has increased during the past decade due to the necessity for production of materials with specific properties for adsorptive processes, with good specific surfaces, good reactivity, and a huge number of active centers on the material surface [9].

The aim of the present study was to prepare a new adsorbent material by functionalization of a cheap and environment friendly commercial polymeric support (Amberlite XAD7) with crown ether (di-benzo-18-crown-6—DB18C6, which has the structure presented in Figure S1 Supplementary Material). Crown ethers are well known for their ability to form complexes with different metallic ions by including them inside of their ring; such complex formation depends on the ring dimension and the metallic ion size. Preparation of tested adsorbent material was done by functionalization of Amberlite XAD7 resin with crown ether by using the solvent-impregnated resin method (SIR method) [10,11,12,13].

The obtained adsorbent material has been used for Pd(II) recovery by adsorption, due to the high complexing capacity of DB18C6 extractant, proving a higher efficiency for Pd(II) removal by adsorption. Adsorptive performance of the newly produced material was demonstrated by kinetics, thermodynamics and equilibrium studies, further leading to a possible adsorption mechanism.

## 2. Materials and Methods

### 2.1. Materials

The support used was Amberlite XAD7 (Sigma-Aldrich, Merck, Darmstadt, Germany), a commercial polymeric resin with acrylic matrix with particles size between 20 and 60 mesh, having a pore volume of 0.5 mL g^−1^ and a specific surface area of 380 m^2^ g^−1^. The extractant used was dibenzo 18-crown-6 (DB18C6) ether, which is a macrocyclic polyether having the IUPAC name 1,4,7,10,13,16-Hexaoxacyclooctadecane and a purity of 99%. Matrix and extractant were purchased from the Sigma-Aldrich company (St. Louis, MO, USA). Amberlite functionalization was achieved by using the solvent-impregnated resin method (SIR method), in which the extractant is dissolved in nitrobenzene (99% purity, purchased from Carl Roth, Karlsruhe, Germany). In order to prepare the Pd(II) aqueous solutions used to establish optimum conditions, we used a stock solution containing 1000 mg Pd(II) L^−1^ in 0.5 mol HNO_3_ L^−1^, purchased from Merck, Darmstadt, Germany.

### 2.2. Characterization and Preparation of Adsorbent

#### 2.2.1. Functionalized of the Amberlite XAD7 Resin

A newly prepared material, designated as XAD7-DB18C6, was obtained through functionalization by impregnation using the dry SIR method, using a mass ratio support:extractant = 10:1. In order to achieve the functionalization of Amberlite resin, the two components were kept in contact for 24 h, after that being dried for 24 h at 323 K.

#### 2.2.2. Characterization of Adsorbent Material

In order to understand the applicability of such materials in adsorptive processes it is important to prove the presence of the extractant on the support surface. So, the presence of crown ether extractant on the surface of the Amberlite obtained material was characterized by scanning electron microscopy (SEM) coupled with X-ray energy dispersive spectroscopy (EDX) using an FEI Quanta FEG 250 scanning electron microscope (FEI, Hillsboro, OR, USA). The presence of DB18C6 crown ether on the functionalized support was confirmed by recording the FT-IR spectra (FTIR, Bruker, Billerica, MA, USA) of the new material. Further new prepared material was characterized by determining its specific surface by using the Brunauer–Emmett–Teller (BET) method, using a Quantachrome Nova 1200e instrument (Anton Paar GmbH, Osfildern-Scharnhausen, Germany).

### 2.3. Effect of Recovery Parameters

The adsorptive performance of the prepared material was highlighted by kinetic, thermodynamic and equilibrium studies, which further led to a possible adsorption mechanism. The influence of specific parameters for the adsorptive processes (such as contact time, temperature, Pd(II) initial concentration) on the maximum adsorption capacity was studied.

In this case, the maximum adsorption capacity of the adsorbent material, q (mg g^−1^) was calculated using the following equation:

(1) $$q=\frac{\left(C_{0}-C_{f}\right)V}{m}$$

where C_0_—initial concentration of Pd(II) from solution, (mg L^−1^)

C_f_—residual concentration of Pd(II) from solution, (mg L^−1^)

V—volume solution, (L)

m—adsorbent mass, (g).

### 2.4. Kinetc Sudies

Contact time and temperature represent important factors used for further evaluation of the newly prepared material for Pd(II) ions. In order to evaluate the influence of contact time and temperature on the maximum adsorption capacity of XAD7-DB18C6 0adsorbent, we weighed 0.1 g of material which was mixed with 25 mL of Pd(II) solution having a concentration of 20 mg Pd(II) per L. Samples were stirred at 200 rotations per minute for different times (30, 60, 90, 120, 180 and 240 min.) at different temperatures (298, 308 and 318 K) using a thermostatic bath (Julabo SW23, Julabo, Seelbach, Baden-Württemberg, Germany). After that the residual concentration of Pd(II) ions was determined by atomic adsorption spectrometry using a Varian SpectrAA 280 FS atomic adsorption spectrometer (Varian, Palo Alto, CA, USA). All experiments were carried out at pH 2.

#### 2.4.1. Kinetic Models

Kinetic models used to describe the studied adsorptive process were obtained by modeling recorded experimental data with two well-known models: the pseudo first-order model—Lagergren model [13,14], described by the equation:

(2) $$\ln\;\left(q_{e}-q_{t}\right)={\mathrm{lnq}}_{e}-k_{1}\;t$$

where q_e_—equilibrium adsorption capacity, (mg g^−1^)

q_t_—adsorption capacity at time t, (mg g^−1^)

k_1_—speed constant for pseudo first-order (min.^−1^)

t—contact time, (min.)

and the pseudo second-order model—Ho and McKay model [13,15,16]:

(3) $$\frac{t}{q_{t}}=\frac{1}{k_{2}\;q_{e}^{2}}+\frac{t}{q_{e}}$$

where q_e_—equilibrium adsorption capacity, (mg/g)

q_t_—adsorption capacity at time t, (mg/g)

k_2_—speed constant for pseudo second-order, (g/mg ∙ min.)

t—contact time, (min.).

When the experimental data were modeled using the pseudo first-order model, the dependence between ln(qe-qt) versus t was calculated. From the obtained straight line equation, we determined the speed constant (k_1_) associated with the model and the maximum adsorption capacity (q_e,calc_). Similarly, for the pseudo second-order model we plotted the linear dependence between t/q_t_ versus t. From the line equation associated with this model were calculated the speed constant (k_2_) and maximum adsorption capacity for the pseudo second-order model.

#### 2.4.2. Activated Energy, E_a_

Another parameter used to characterize the adsorption process of Pd(II) onto different adsorbent materials is the activation energy (E_a_). This parameter can be evaluated from the following Arrhenius equation:

(4) $$\ln\;k_{2}=\mathrm{lnA}-\frac{E_{a}}{\mathrm{RT}}$$

where k_2_—kinetic constant obtained from the pseudo second-order model, (g min^−1^ mg^−1^)

A—Arrhenius constant, (g min^−1^ mg^−1^)

E_a_—activation energy, (kJ mol^−1^)

T—absolute temperature, (K)

R—the ideal gas constant, (8.314 J mol^−1^ K^−1^).

The activation energy for adsorption of Pd(II) is calculated from the linear dependence of lnk_2_ versus 1/T.

#### 2.4.3. Thermodynamic Parameters

In order to confirm that the Pd(II) adsorption on the XAD7-DB18C6 adsorbent is a spontaneous process, we determined the value of free Gibbs energy (ΔG^0^) by using the Gibbs–Helmholtz equation [17]:

(5) $$\Delta G^{0}=\;\Delta H^{0}-T\;\Delta S^{0}$$

where ΔG^0^—the standard variation of Gibbs free energy (kJ/mol)

ΔH^0^—the standard variation of enthalpy, (kJ/mol)

ΔS^0^—the standard variation of entropy, (J/mol ∙ K)

T—absolute temperature, (K).

Entropy standard variation (ΔS^0^) and enthalpy standard variation (ΔH^0^) were determined using the van’t Hoff Equation (6), by plotting the linear dependence between lnK_d_ versus 1/T.

(6) $$\ln\;K_{d}=\frac{{\Delta S}^{\circ }}{R}-\frac{{\Delta H}^{\circ }}{\mathrm{RT}}$$

where K_d_—equilibrium constant

ΔS^0^—the standard variation of entropy, (J mol^−1^ K^−1^)

ΔH^0^—the standard variation of enthalpy, (kJ mol^−1^)

T—absolute temperature, (K)

R—the ideal gas constant, (8.314 J mol^−1^ ∙ K^−1^).

The equilibrium constant is ratio between the adsorption capacity at equilibrium q_e_ and the equilibrium concentration C_e_:

(7) $$K_{d}=\frac{q_{e}}{C_{e}}$$

where q_e_—equilibrium adsorption capacity, (mg/g)

C_e_—equilibrium concentration, (mg/L).

### 2.5. Equilibrium Studies

For equilibrium studies, 0.1 g of XAD7-DB18C6 adsorbent material was mixed with 25 mL of Pd(II) solution having different concentrations (5, 10, 15, 20, 30, 40, 50, 60, 70 and 80 mg L^−1^). All adsorption studies were carried out for 1 h, pH 2, at 298 K by using a thermostatic bath. At the end of each experiment, each sample was filtered. In each obtained solution we determined the residual concentration of Pd(II) ions.

#### Adsorption Isotherms

Establishing the equilibrium data, which are generally known as adsorption isotherms, represents a basic requirement for further understanding of the adsorption mechanism. Classic adsorption isotherms used to describe adsorption processes are Langmuir, Freundlich and Sips, which were also used in the present study to describe the adsorption process of Pd(II) ions onto XAD7-DB18C6.

The Langmuir adsorption isotherm is used to describe the adsorptive processes in homogenous media, explaining well monolayer adsorptive processes. In the case of the Langmuir adsorption isotherm, active centers are identical and evenly distributed on the adsorbent surface, and the ability of a molecule to adsorb on one of the active centers is independent of the occupancy of the neighboring active centers. The nonlinear form of the Langmuir adsorption isotherm is [18]:

(8) $$q_{e}=\frac{q_{L}\;K_{L}\;C_{e}}{1+K_{L}\;C_{e}}$$

where q_e_—equilibrium adsorption capacity, (mg g^−1^)

C_e_—equilibrium concentration for Pd(II) ions from solution, (mg L^−1^)

q_L_—Langmuir maximum adsorption capacity, (mg g^−1^)

K_L_—Langmuir constant.

One important characteristic of the Langmuir isotherm is the dimension less constant R_L_, named the separation factor. This parameter can be evaluated with the equation:

(9) $$R_{L}=\frac{1}{1+K_{L}\;\mathrm{Co}}$$

where R_L_—separation factor;

K_L_—Langmuir constant (L mg^−1^)

C_o_—Pd(II) initial concentration, (mg L^−1^).

The Freundlich isotherm [19] assumes that the adsorbent surface is heterogenous, on which the distribution of adsorption heat is nonuniform, and a multilayer adsorption can occur due to the unlimited number of active centers. The nonlinear form of the Freundlich isotherm is:

(10) $$q_{e}=K_{F}\;C_{e}^{1/n_{f}}$$

where q_e_—equilibrium adsorption capacity, (mg g^−1^)

C_e_—equilibrium concentration for Pd(II) from solution, (mg g^−1^)

K_F_ and n_F_—characteristic constants which may be associated with the relative adsorption capacities in respect of the adsorption intensity.

Starting from these two adsorption isotherms, we developed the Sips isotherm, which is used to explain adsorptive processes. At low concentrations, the Sips isotherm is reduced at the Freundlich isotherm, and at higher concentration of adsorbate it is reduced at the Langmuir isotherm; therefore, it can be used to calculate the monolayer adsorption capacity. The nonlinear form of the Sips isotherm is [20]:

(11) $$q_{e}=\frac{q_{S}\;K_{S}\;C_{e}^{1/n_{S}}}{1+K_{S}\;C_{e}^{1/n_{S}}}$$

where q_S_—maximum equilibrium adsorption capacity (mg g^−1^)

K_S_—constant related to the adsorption capacity of the adsorbent

n_S_—heterogenic factor.

## 3. Results and Discussion

### 3.1. Characterization of the Adsorbents

#### 3.1.1. Scanning Electron Microscopy (SEM)

**Figure 1:**
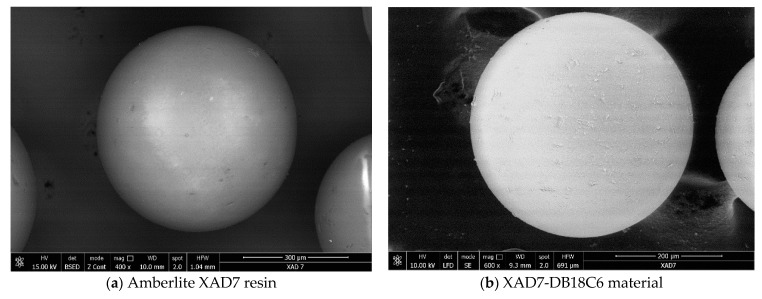
Scanning electron microscopy (SEM) before (**a**) and after functionalization (**b**).

In Figure 1 are depicted the SEM micrographs recorded for Amberlite XAD7 support before and after functionalization with DB18C6 crown ether.

From the recorded micrographs, we can observe the presence of some white dots on the support surface in case of functionalized Amberlite XAD7 resin. The presence of these white spots can be associated with the presence of DB18C6 crown ether molecules on the support surface.

#### 3.1.2. X-Ray Dispersive Energy Spectroscopy (EDX)

**Figure 2:**
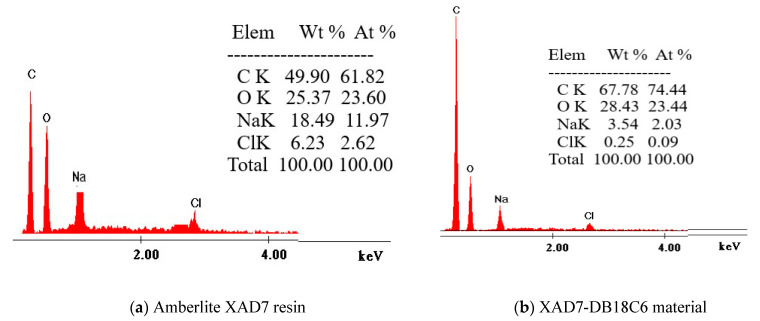
X-ray dispersive energy spectroscopy (EDX) before (**a**) and after functionalization (**b**).

X-ray dispersive energy spectroscopy (EDX) was used to obtain information regarding the elemental composition of analyzed samples, the obtained data being depicted in Figure 2.

**Figure 3:**
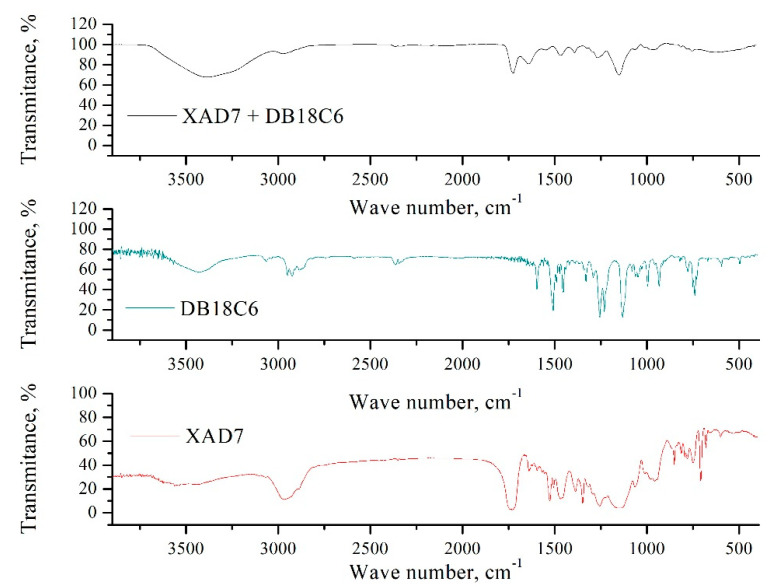
FT-IR spectra recorded for Amberlite XAD7 and Amberlite XAD7-DB18C6.

From data depicted in Figure 3, we can observe that the carbon and oxygen concentrations increased in case of functionalized support, being a confirmation of functionalization of Amberlite XAD7 with DB18C6 crown ether.

#### 3.1.3. Fourier Transform Infrared Spectroscopy (FT-IR)

Further confirmation of Amberlite XAD7 functionalization was obtained from FT-IR spectra recorded for pure and functionalized support (spectra are depicted in Figure 3).

From the FT-IR spectra of Amberlite XAD7, we can observe a broad band located at 3450 cm^−1^ which is associated with the stretching vibrations of H-O groups. Sharp and strong adsorption bands located at 2975, 2930, 2890 cm^−1^ are associated with the stretching vibrations of the C-H bond. The vibration peak observed at 1745 cm^−1^ can be associated with the vibrations of –C=O groups, and the vibrations from 1477 and 1390 cm^−1^ are attributed to stretching and deformation of the C-H bond from –CH_3_ aliphatic groups. Vibrations observed at 1260 and 1135 cm^−1^ are associated with the stretching vibrations of –C-O groups [21].

Amberlite XAD7 functionalization with DB18C6 crown ether induced some modifications in the FT-IR spectra; modifications which are associated with the presence of DB18C6 crown ether on the support surface. From Amberlite XAD7-DB18C6 spectra, we can observe the presence of a vibration at 3017 cm^−1^; a vibration which can be associated with the stretching of the C-H bond from the aromatic nucleus. Another different vibration can be observed at 2938 cm^−1^, which is associated with the stretching of the C-H bond from methylene groups. This vibration becomes more pronounced for functionalized Amberlite XAD7. A specific vibration for DB8C6 crown ether can be observed at 1302 cm^−1^; a vibration which can be associated with the symmetric stretching of the Ph-O-C group, followed by the appearance of a non-symmetric vibration at 1240 cm^−1^. Another specific vibration appeared at 1128 cm^−1^; this vibration can be associated with the symmetric stretching of free C-O-C groups [22].

#### 3.1.4. BET Surface Area

**Figure 4:**
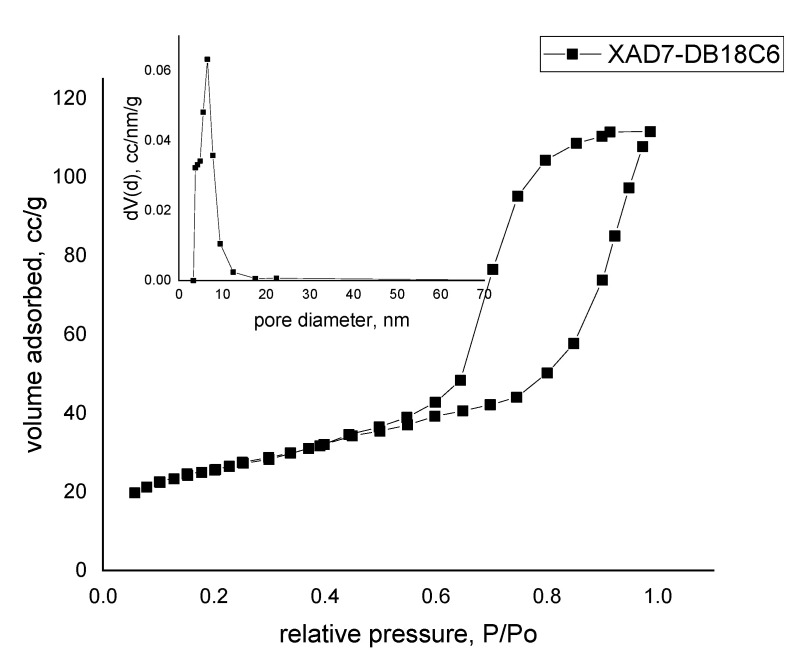
Brunauer–Emmett–Teller (BET) surface area.

The N_2_ adsorption-desorption isotherms were obtained by using a Quantachrome NOVA 1200e device. The samples were degassed beforehand at room temperature in a vacuum for 4 h. The analysis was done at 77 K with nitrogen atmosphere. In Figure 4 is presented the experimental data point of the sample with pore size distribution (inset picture).

Evaluating the data with IUPAC [17], we can conclude that the material presenting a type IV isotherm was obtained. The presence of hysteresis is one of the main factors that can show the occurrence of capillary condensation. Comparing the hysteresis obtained with IUPAC, we obtained a type H2b representative for pore blocking, but the size distribution of neck widths was now much larger.

The BET method (Brunauer–Emmett–Teller) was used in order to obtain the surface area in the range 0.05–0.30 P/Po, indicating a value of 92 m^2^ g^−1^. The total pore volume was obtained from the last point of isotherm at P/Po = 0.98655, indicating a value of 1.730 × 10^−1^ cc g^−1^ for pores smaller than 145.0 nm. Using the BJH method (Barrett–Joyner–Halenda) [23] from the desorption branch, the pore size distribution indicated a unimodal distribution in the mesoporous region with a mean value of ~6.5 nm, also represented in inset Figure 4.

### 3.2. Effect of Recovery Parameters

#### 3.2.1. Contact Time and Temperature Influence

**Figure 5:**
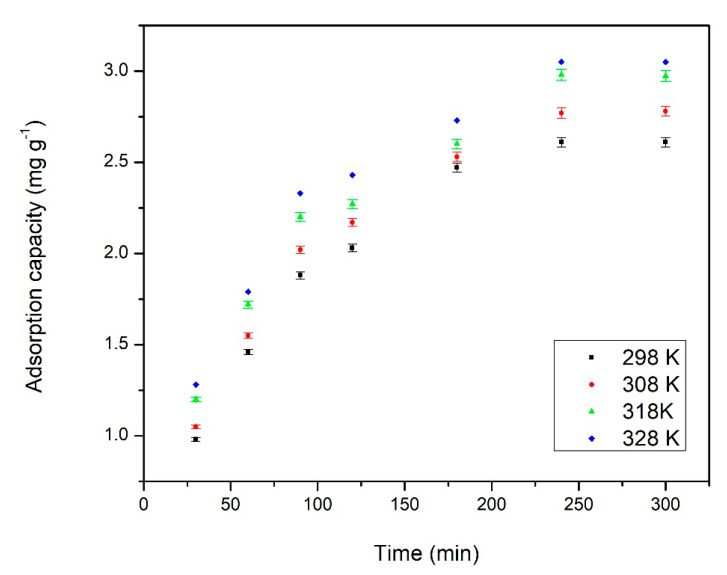
Influence of contact time and temperature on the adsorption capacity of Pd(II) on XAD7-DB18C6.

In order to better understand adsorptive processes, it is important to know the contact time and temperature needed to reach adsorbent—adsorbate equilibrium. In Figure 5 is depicted the influence of contact time at four different temperatures (298, 308, 318 and 328 K) obtained for Pd(II) adsorption onto Amberlite XAD7-DB18C6.

**Figure 6:**
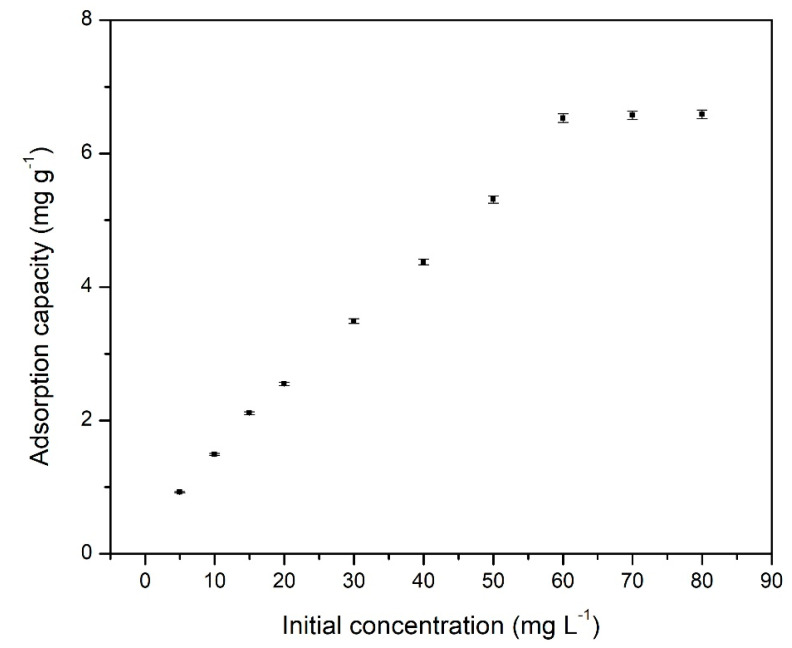
Influence of initial concentration for Pd(II) recovery on XAD7-DB18C6.

From the data depicted in Figure 6, we can observe that with the increase of the contact time an increase of adsorption capacity occurred, until the 240 min. mark. Further increase of the contact time led to no significant increase of the adsorption capacity. Based on this observation, we can consider that 240 min. represented the time needed for the studied system to reach equilibrium. Further experiments were carried out using a contact time of 240 min.

From data depicted in Figure 6, we can also observe that temperature had a great influence over the Pd(II) adsorption onto Amberlite XAD7-DB18C6. The increase of temperature from 298 to 328 K led to an increase of adsorption capacity from 0.83 to 3.05 mg g^−1^.

#### 3.2.2. Pd(II) Initial Concentration Influence

Distribution of Pd(II) ions between adsorbent material and aqueous solution at equilibrium assumes a higher importance for further determination of maximum adsorption capacity at equilibrium [24].

The aim of the present study was to determine the maximum adsorption capacity of Amberlite XAD7—DB8C6 (data being depicted in Figure 6).

From experimental data presented in Figure 6, we can observe that the increase of Pd(II) initial concentration led to an increase of the quantity of Pd(II) ions adsorbed onto the Amberlite XAD7—BD18C6 crown ether. Such increase is due to the presence of free active sites on the material surface; when these active sites were occupied, any further increase of Pd(II) initial concentration led to no increase of maximum adsorption capacity of the material. In case of the studied adsorption process, the maximum adsorption capacity was 6.5 mg g^−1^, and was reached at an initial concentration of Pd(II) ions equal to 60 mg L^−1^.

### 3.3. Adsorption Kinetics

**Figure 7:**
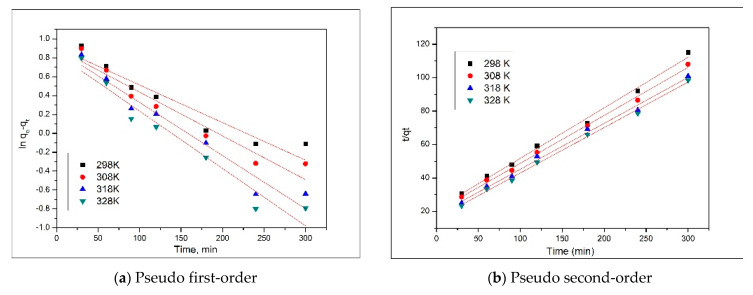
Kinetic models for Pd(II) adsorption onto XAD7-DB18C6, at different temperatures.

Kinetics of the adsorptive processes are dependent on the interactions established between adsorbate and adsorbent material. To assess the kinetic mechanism of the Pd(II) adsorption onto Amberlite XAD7-DB18C6 material, all experimental data were modeled using pseudo first-order and pseudo second-order kinetic equations. Obtained linear dependences are presented in Figure 7, and based on depicted data we evaluated the parameters associated with these two models (presented in Table 1).

The value of the k_1_ constant—associated with the pseudo first-order model—was evaluated from the slope of the linear dependence ln(q_e_ − q_t_) versus time. Similarly, we evaluated the value of the k_2_ constant—associated with the pseudo second-order model—from the slope of linear dependence t/q_t_ versus time. The studied adsorption process was better described by the model for which the correlation coefficient was much closer to 1. Based on data presented in Table 1, we can observe that the Pd(II) adsorption onto Amberlite XAD7-DB18C6 was better described by the pseudo second-order model, for which the correlation coefficient was located between 0.9950 and 0.9971, depending on the temperature. This correlation is in concordance with the literature data, showing that the Pd(II) adsorption was influenced by time and temperature [1,25].

For the studied adsorption process, it seems that the chemical reactions show the limiting step of the adsorption process is of great importance [26].

**Figure 8:**
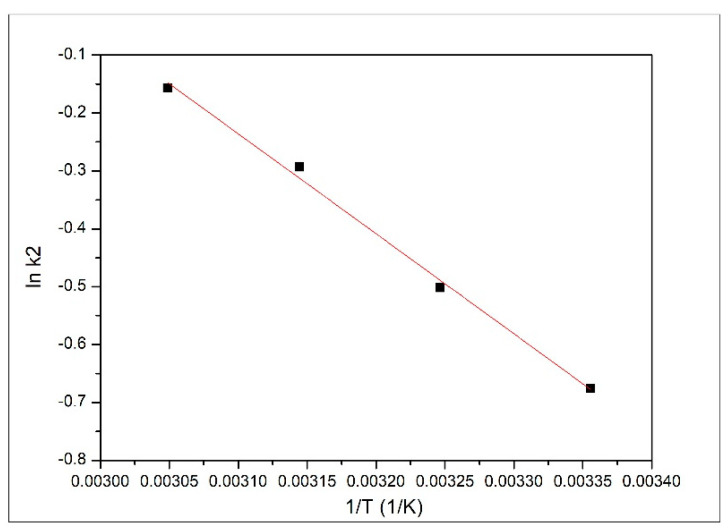
Arrhenius plot.

Further, by using the speed constant obtained from pseudo second-order model (k_2_) and the Arrhenius equation, we calculated the activation energy associated with the studied adsorption process. The value of the activation energy was calculated from the slope of the linear dependence between lnk_2_ and 1/T (data depicted in Figure 8).

Based on the obtained experimental data, we determined a value of 1.44 kJ mol^−1^ for the activation energy, with a correlation coefficient of 0.9960. If the activation energy has a value lower than 40 kJ mol^−1^, we can say that the Pd(II) ions’ adsorption is a physical adsorption [27].

### 3.4. Adsorption Equilibrium

**Figure 9:**
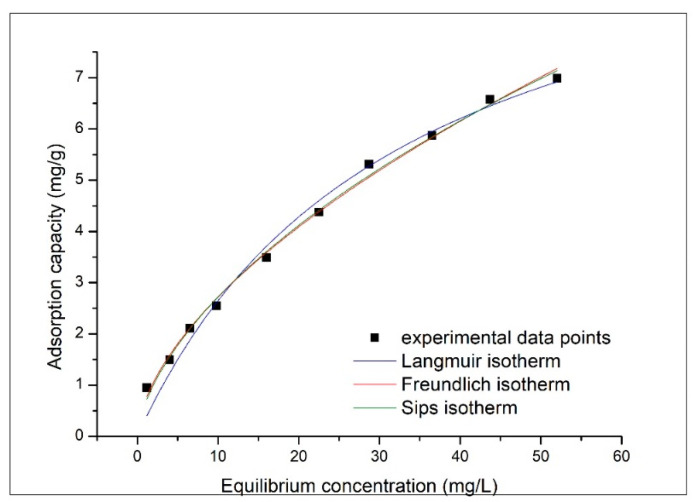
Adsorption isotherm model for adsorption of Pd(II) onto XAD7-DB18C6.

In order to understand the Pd(II) ions’ behavior at the interface during the adsorption process, obtained experimental data were modeled using three isotherms: Langmuir, Freundlich and Sips. The correlation coefficient, R^2^, was determined for each isotherm to establish which one better described the Pd(II) adsorption onto Amberlite XAD7-DB18C6 adsorbent material. Obtained adsorption isotherms are shown in Figure 9 and, based on data presented in this figure, we determined the parameters specific to each isotherm (Table 2).

Based on data presented in Table 2, we can observe that the correlation coefficient had the biggest value in case of the Sips isotherm (0.99531) and was closer to unity, meaning that this isotherm better described Pd(II) ions’ adsorption onto the studied adsorbent. It can also be observed that the maximum value of adsorption capacity evaluated based on the Sips isotherm was 5.9 mg g^−1^, much closer to the experimental one 6.5 mg g^−1^. Based on the value of the coefficient ns, which is higher than 1, we can say that the studied adsorption process is a heterogeneous one.

In Table 3 are presented some other synthesized or modified adsorbent materials used for Pd(II) recovery from aqueous solutions. The well-known ability of Amberlite XAD7 resin, and especially the ability of functionalized Amberlite XAD7, in the recovery of metallic ions from aqueous solutions is in concordance with data from the literature [28,29] and was also confirmed by experimental data obtained in the present study.

### 3.5. Thermodynamic Parameters

**Figure 10:**
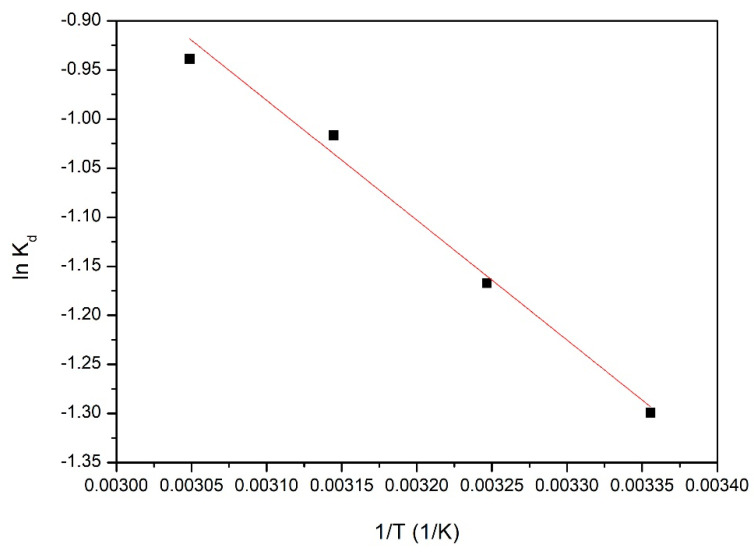
Plot of ln K_d_ vs 1/T for the estimation of thermodynamic parameters for Pd(II) recovery on XAD7-DB18C6.

To investigate the spontaneity of the studied adsorption process, we determined the thermodynamic parameters from the linear dependence between lnk_d_ and 1/T (graph depicted in Figure 10). Obtained thermodynamic parameters are presented in Table 4.

Based on the obtained experimental data, we evaluated the values of thermodynamic parameters—free Gibbs energy (ΔG°), enthalpy (ΔH°) and entropy (ΔS°). At the same time, the regression coefficient, R^2^, value was established. The positive value of the enthalpy, ΔH°, demonstrated that the energy required for the adsorption process was the energy used to put in contact Pd(II) ions with the surface of the adsorbent material. Adsorption of Pd(II) ions onto the adsorbent surface was possible due to electrostatic attraction and may be due to endothermic complexation processes.

The value of the free Gibbs energy, ΔG°, calculated from the experimental data was negative, indicating that the adsorption of Pd(II) on XAD7- DB18C6 was a spontaneous process. This value became more negative as the temperature increased, which can be attributed to the effective increase of the contact surface between the adsorbent material and the Pd(II) ions.

The positive value of entropy (ΔS°) suggests that adsorption speed increased at material/solution interface and the degree of particle clutter increased with increasing temperature, which can be attributed to changes at the surface of the material. Thus, the adsorption of Pd(II) onto the material surface was an endothermic and spontaneous process.

The aqueous solution at pH ~3 Pd can be expressed in the form of [Pd(NO_3_]_4_]^2−^. Based on these considerations, the following mechanism is proposed:

(12) $${2H}_{\mathrm{aq}}^{+}+[\mathrm{Pd}\left({\mathrm{NO}}_{3}\right){]}_{4}{]}_{\mathrm{aq}}^{2-}{+S}_{s}{=[(H}^{+}{)}_{2}{{(\mathrm{Pd}(\mathrm{NO}}_{3}{)}_{4})}^{2-}{]}_{s}$$

where aq and s represent the aqueous or solid phase. Ss is the solid support of Amberlite XAD7-DB18C6 resin.

The proposed mechanism is also confirmed by the literature data [5].

## 4. Conclusions

In the present study, a new adsorbent material was obtained by chemical modification of Amberlite XAD7 polymer by functionalization with dibenzo-18-crown-6 ether. Presence of the crown ether on the polymer surface was evidenced by the characterization of the obtained material by X-ray energy dispersion (EDX), scanning electron microscopy (SEM) and Fourier transform infrared spectroscopy (FT-IR). At the same time, the specific surface of the Amberlite support was determined, as was the material obtained after functionalization by impregnation with crown ether. It was observed that the extractant migrated into the resin pores, reducing its specific surface.

The adsorption of Pd(II) ions onto the XAD7-DB18C6 material was spontaneous, endothermic and can be considered due to physical processes that took place at the adsorbent-adsorbed interface.

Experimental data were better modeled by the pseudo second-order kinetic model and by the Sips adsorption isotherm. Evaluation of the thermodynamic parameters from the van’t Hoff equation indicated that the studied adsorption process was a spontaneous and endothermic one. The newly produced adsorbent material (XAD7-DB18C6) had a maximum adsorption capacity of 6.5 mg Pd(II) per g of adsorbent, representing a possible candidate for recovery of Pd(II) ions from residual solutions.

## Figures and Tables

**Table 1:** Kinetic parameters for adsorption of Pd(II) onto XAD7-DB18C6 material.

**Pseudo First-Order**				
Temperature (K)	q_e,exp_	k_1_	q_e,calc_	R^2^
	(mg · g^−1^)	(min^−1^)	(mg · g^−1^)	
298	2.61	0.004 ± 0.00001	2.48 ± 0.01	0.9139 ± 0.001
308	2.77	0.0046 ± 0.00001	2.46 ± 0.01	0.9401 ± 0.001
318	2.98	0.0056 ± 0.00001	2.43 ± 0.01	0.9548 ± 0.001
328	3.05	0.0061 ± 0.00001	2.32 ± 0.01	0.9593 ± 0.001
**Pseudo Second-Order**				
Temperature (K)	q_e,exp_	k_2_	q_e,calc_	R^2^
	(mg · g^−1^)	(g · mg^−1^ ∙ min^−1^)	(mg · g^−1^)	
298	2.61	0.508 ± 0.00001	3.30 ± 0.01	0.9950 ± 0.001
308	2.77	0.605 ± 0.00001	3.44 ± 0.01	0.9971 ± 0.001
318	2.98	0.746 ± 0.00001	3.64 ± 0.01	0.9949 ± 0.001
328	3.05	0.855 ± 0.00001	3.67 ± 0.01	0.9967 ± 0.001

**Table 2:** Parameters of isotherm model for adsorption of Pd(II) onto XAD7-DB18C6.

**Langmuir Isotherm**			
q_m,exp_ (mg g^−1^)	K_L_ (L mg^−1^)	q_L_ (mg g^−1^)	R^2^
6.5	0.0308 ± 0.01	3.2 ± 0.01	0.98549 ± 0.001
**Freundlich Isotherm**			
K_F_ (mg g^−1^)	1/n_F_		R^2^
0.7001	0.589 ± 0.01		0.97164 ± 0.001
Sips Isotherm			
K_S_	q_S_ (mg g^−1^)	1/n_S_	R^2^
0011	5.9 ± 0.01	0.36 ± 0.01	0.99531 ± 0.001

**Table 3:** Comparison to other materials for Pd(II) adsorption.

Adsorbents	Adsorption Capacities, mg g^−1^	Reference
Crosslinked carboxymethyl chitosan hydrogels	1.05	[30]
Fungus aspergillus sp. immobilized on Cellex-T	0.47	[31]
Thiocyanate retaining tannin gel	6.92	[32]
AP-XAD 16 resin	8	[33]
Unmodified chitosan	5.9	[34]
Polystyrene divinylbebzene-Dithiooxamide	10.6	[35]
Cellulose-MBT	5	[36]
Native cellulose	1.87	[36]
Ion exchange resin Diaion WA21J	4.85	[37]
Radiation cross-linked carboxymethylchitosan hydrogels	1.063	[30]
XAD7-DB18C6	6.5	Present paper

**Table 4:** Thermodynamic parameters for adsorption of Pd(II) onto XAD7-DB18C6.

ΔH° (kJ mol^−1^)	ΔS° (J mol^−1^ ∙ K^−1^)	ΔG° (kJ mol^−1^)				R^2^
10.16	23.3	298 K	308 K	318 K	328 K	0.9912
		−6.94	−7.17	−7.40	−7.63	

## Data Availability

Data will be available at request.

## Supplementary Materials

The following are available online at https://www.mdpi.com/1996-1944/14/4/1003/s1. Figure S1. Dibenzo-18-crown-6 ether structure.
