# Supplementary figures and images for: Evaluation of Performance of Functionalized Amberlite XAD7 with Dibenzo-18-Crown Ether-6 for Palladium Recovery
Source: Materials (Basel). 2021 Feb 20;14(4):1003. doi: 10.3390/ma14041003 (PMC7924212; doi:10.3390/ma14041003)

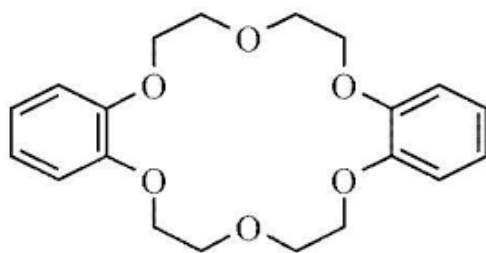

**Figure S1.** Dibenzo-18- crown-6 ether structure.

Supplement: Supplementary file 1 [file materials-14-01003-s001.pdf]
